# The space of words: semantic integration of space-related words in noun sensorimotor processing

**DOI:** 10.1007/s00426-026-02326-1

**Published:** 2026-08-08

**Authors:** Gioacchino Garofalo, Lucia Riggio, Francesco Bianchini, Elena Gherri

**Affiliations:** 1 Department of Philosophy, University of Bologna, Via Zamboni 38, Bologna, 40126 Italy; 2 Department of Medicine and Surgery- Unit of Neuroscience, University of Parma, Via Volturno 39, Parma, 43125 Italy

## Abstract

Evidence suggests that the processing of graspable object nouns elicits specific motor programs related to potential hand-object interactions. Notably, adjectives specifying manipulative features of these objects are integrated into this sensorimotor representation. In the present study, three experiments were conducted to explore the impact of spatial adjectives denoting the position of the object in space on the sensorimotor representation of graspable object nouns. We used a reach-to-grasp compatibility task, in which participants had to categorize object nouns as artifact or natural, by performing either a power or a precision reach-to-grasp movement matching or not the typical grip associated with the object. On each trial the object noun was presented with a second word specifying the location of the object in space (e.g. near vs. far). In addition, using the same task, we explore also the impact of a different class of words on the object sensorimotor representation that are spatial demonstratives (i.e., this and that) In all experiments, a reliable grasp compatibility effect emerged. The grasp-compatibility effect was modulated differentially by spatial adjectives and spatial demonstratives, revealing not only that both elements are integrated into the noun sensorimotor representation, but also that these different word categories lead to different spatial representations of the object during the semantic processing of object-related words combinations.

## Introduction

Affordances are defined as the possibilities for action/interaction offered by objects in the environment to a given organism, given its biological constraints (Gibson, 1979). This definition was further specified by Ellis and Tucker (2000) with the introduction of “micro-affordances”, according to which perceiving specific objects features (e.g., size) potentiates specific action components (e.g., grip). Micro-affordances have been investigated using the grasp-compatibility task in which participants had to categorize an object as natural or artifact by grasping a response device with a power or a precision grip. Performance improves when the response grip is congruent with the object size (e.g., small object and precision grip, compatible trials), while it worsens when the response grip is not congruent (e.g., small object and power grip, incompatible trials). This difference in performance, referred to as the grasp-compatibility effect, suggests that seeing a graspable object activates a set of potential hand motor programs associated with the pragmatic characteristic of the object (e.g., size and shape), facilitating the execution of the congruent response (e.g., Bub et al., 2015; Derbyshire et al., 2006; Garofalo & Riggio, 2022; Tucker & Ellis, 2004).

Micro-affordances derive from perception-action patterns of hand–object interaction represented in the brain (Ellis & Tucker, 2000; Grèzes et al., 2003). These patterns can be defined as ‘*stable’* affordances when they emerge from consolidated and constant experiences across different contexts, as for example the typical way in which we grasp objects with different sizes. Conversely, affordances can be defined as ‘*variable’* when they are related to temporary pragmatic object properties, such as its orientation or spatial location, requiring an online updating of information to define the current state of the object. Given the variability of these properties across contexts, variable affordances are not usually stored in the object representation (Borghi and Riggio 2009, 2015). Objects spatial features are crucial for our sensorimotor system to parametrize object-directed actions (Jeannerod et al., 1995). In everyday life, we typically interact with objects within a well-defined portion of space. This sector of space includes objects that are located near enough to be reached by our effectors, that is the peripersonal space (PPS). Objects located within PPS are within hand-reaching distance whereas objects located outside PPS, that is in extrapersonal space (EPS), cannot be reached without approaching movements of the whole body (Berti and Frassinetti 2000; di Pellegrino and Làdavas 2015). Increasing evidence has demonstrated that primates have different brain representations for different sectors of space (e.g., Berti & Frassinetti, 2000; Gross & Graziano, 1995; Maravita et al., 2003). For example, physiological evidence showed that PPS and EPS recruit two separate fronto-parietal circuits designed to solve different computational problems involving motor control. It has been proposed that these sectors of space are encoded separately by the brain in terms of potential motor acts (Fogassi et al., 1996; Rizzolatti et al., 1997). The representation of PPS is mediated by the same circuit involved in the visuomotor control of arm and hand movements, while circuits controlling for eye movements and gaze direction are the same involved in coding the EPS (for a review Gross & Graziano, 1995). Accordingly, behavioral studies, using different tasks, demonstrated that graspable objects elicit motor-related effects when an object is presented in PPS, but not when presented beyond reach where the involvement of the hand motor system is reduced (Ambrosini & Costantini, 2013; Costantini et al., 2010, 2011; De Stefani et al., 2014; Kalénine et al., 2016). In particular, Costantini et al. (2010, 2011) conducted studies involving a 3D hand-to-handle alignment task. Participants were asked to mimic a reach-to-grasp movement, either with the left or the right hand, based on a given instruction. The researchers found that participants responded faster when the orientation of their responding hand matched the orientation of the virtual object, but only if the object was positioned within a reachable area of the 3D environment.

Recent evidence has demonstrated that also the processing of graspable objects nouns leads to the activation of specific motor programs (for review see, Buccino et al., 2016; Vigliocco et al., 2011), shown by the presence of grasp-compatibility effects related to the object size denoted by the noun (e.g., Bub & Masson, 2012; Garofalo et al., 2021; Glover et al., 2004; Marino et al., 2013). Thus, stable affordances are re-enacted during noun processing (Borghi & Riggio, 2015). By contrast, the processing of temporary object features (i.e., variable affordances) does not seem to influence the performance during language comprehension. In two experiments, Ferri and colleagues (2011) investigated whether objects and their nouns share similar motor representations. In Experiment 1, participants performed a grasp-compatibility task, in which they had to categorize 3D objects images as artifact or natural. The objects could be presented in PPS or EPS. Results revealed a grasp-compatibility effect only when the object was placed in PPS. In Experiment 2, participants had to perform the grasping response after determining whether an object image matched a previously presented object noun. Importantly, in this task the grasp-compatibility effect was not modulated by the near or far position of the object. These initial findings suggest that both stable and variable affordances appear to modulate sensorimotor activation driven by objects images, while mainly stable affordances are re-enacted during language processing (Borghi & Riggio, 2015).

Sensorimotor effects elicited by objects nouns differ from those evoked by their visual depictions not least because visual images are characterized by analog information, which is completely lacking in verbal stimuli (e.g., Buccino et al., 2005). In this respect, adjectives, providing further information about the (im)possibility to interact with the object denoted by the noun, can modulate the sensorimotor activation. For example, adjectives that reduce or prevent the possibility to interact with an object (e.g., broken bottle) affect the hand-object motor programs, inhibiting the activation of grasping affordances (Garofalo et al., 2021; see also Gough et al., 2013). Moreover, color adjectives modulate the grasp-compatibility effect differentially for artifact and natural objects. The grasp-compatibility effect is reduced when color adjectives are presented with artifact nouns, while it is increased when color adjectives are presented with natural object nouns (Garofalo et al., 2021; 2024). Intriguingly, the modulatory effects of adjectives on the grasp-compatibility effect disappear when the canonical noun-adjective relative position is reversed (Garofalo et al., 2024). This corpus of evidence strongly suggests that the meaning of adjectives denoting stable object characteristics are elaborated together with the meaning of the noun.

When we look at an object, information about its location in space or its distance from us is immediately available and automatically encoded by the visual system. Conversely, when we read or hear the noun of an object, no spatial information is available, unless other words are used to specify this property. Two different classes of words can be used to describe the location in space of verbally described objects: spatial demonstratives (i.e., this and that) and spatial adjectives (e.g., near and far). While there is mixed evidence about the relationship between spatial demonstratives and the reach-to-grasp actions (e.g., Caldano & Coventry, 2019; Coello & Bonnotte, 2013; Coventry et al., 2008; Rocca et al., 2019), to the best of our knowledge, there is no evidence on the effect of spatial adjectives on the sensorimotor processes elicited by graspable object nouns. Hence, it remains unknown whether adjectives conveying spatial information modulate reach-to-grasp compatibility effects elicited by objects nouns. Here we sought to clarify and expand the existing literature by investigating whether the information carried by spatial adjectives and spatial demonstratives - placing the object in near reachable space (PPS) or beyond it (EPS) – is sufficient to modulate the involvement of the sensorimotor systems during the semantic processing of these words (noun-adjective and demonstrative-noun combinations).

In the present study, we used a well-established reach-to-grasp compatibility task in which participants categorized object nouns as artifact or natural by performing a precision or a power grasp, compatible or not with the size of the object noun. We combined these graspable object nouns with spatial adjectives (Experiment 1, 2 and 3) and with spatial demonstratives (Experiment 2) to investigate if and how the spatial information specified by these different word categories modulated the motor program evoked by the nouns, similarly to what observed in studies presenting visual objects within or outside PPS (e.g., Costantini et al., 2010, 2011).

## Experiment 1

In Experiment 1, participants were instructed to respond to the category of a graspable object noun (natural vs. artifact) with a power or a precision grasp. This response could be either compatible or incompatible with that object (e.g. a power grasp is compatible with the action usually carried out on an apple but not on an olive). Importantly, on each trial the object noun was presented with a spatial adjective specifying the near vs. far location of the object (for example ‘mela vicina’ = near apple, ‘oliva lontana’ = far olive, etc.). Because objects in PPS but not EPS can be grasped and manipulated, one may expect to observe the grasp-compatibility effect related to the size of the object exclusively when the adjective locates the object within reachable space, in line with the spatial modulation of affordances observed for visual depictions of objects (see Costantini et al., 2010, 2011). However, it is also conceivable that the spatial adjective is not integrated in the sensorimotor representation driven by the noun because it denotes a relatively broad sector of space rather than a specific physical position. In other words, it’s possible that the spatial adjective has little impact on the overall grasp-compatibility effect related to the size of the object driven by the noun because it refers to a variable feature of the object. To obtain a fine-grained picture of the impact of spatial adjectives on the grasp-compatibility effect we analyzed the time course of participants’ responses by means of a bin analysis, in addition to their response time analysis, because grasp-compatibility effects related to sensorimotor processing need time to emerge (Ambrosecchia et al. 2015; Bub et al. 2018; Bub and Masson 2012; Garofalo et al. 2020, 2022).

## Methods

### Sample size estimation

We conducted an a-priori power analysis to determine the adequate sample size. We used previously published data (Garofalo et al., 2021 - Experiment 2), obtained with the same task. To determine the sample size, we used the means of trimmed and transformed back data of 26 participants related to the critical two-way interaction (Adjective X Grasp compatibility). The observed power was about 99% for 26 participants in Experiment 2 of Garofalo et al. (2021). Here, we assessed the minimum sample size needed to detect 80% of power involving the same effect size as in Garofalo et al. (2021, Exp. 2). Power analyses were performed using the simr package in R (Green & Macleod, 2016), simulating and then modelling 1000 independent experiments with increasing numbers of participants. Power analyses results showed that the minimum sample size ranged between 14 (Power: 77.80%, 95% CIs = 75.10% − 80.34%) and 16 participants (Power: 84.40%, 95% CIs = 82.00% − 86.66%). We chose to enlarge the sample size to 26 participants since we reduced the number of trials and thus to account for the Type II error.

### Participants

Twenty-six participants (14 identifying as female; 12 identifying as male; mean age in years = 26.4 ± 4.3) volunteered to take part in the experiment at the University of Bologna, but two of them were later excluded from the analyses due to equipment malfunctioning. All participants were right-handed, as measured by a standard handedness inventory (Oldfield, 1971), native-Italian speakers, and had normal or corrected-to-normal vision. All of them were 18 years old, or older, at the time of the study and naïve as to the purpose of the experiment. Participants gave their informed consent before participation, following the ethical standards of the Declaration of Helsinki. The study was approved by the local ethical committee (approval number: 0026467).

### Apparatus and stimuli

**Fig. 1:**
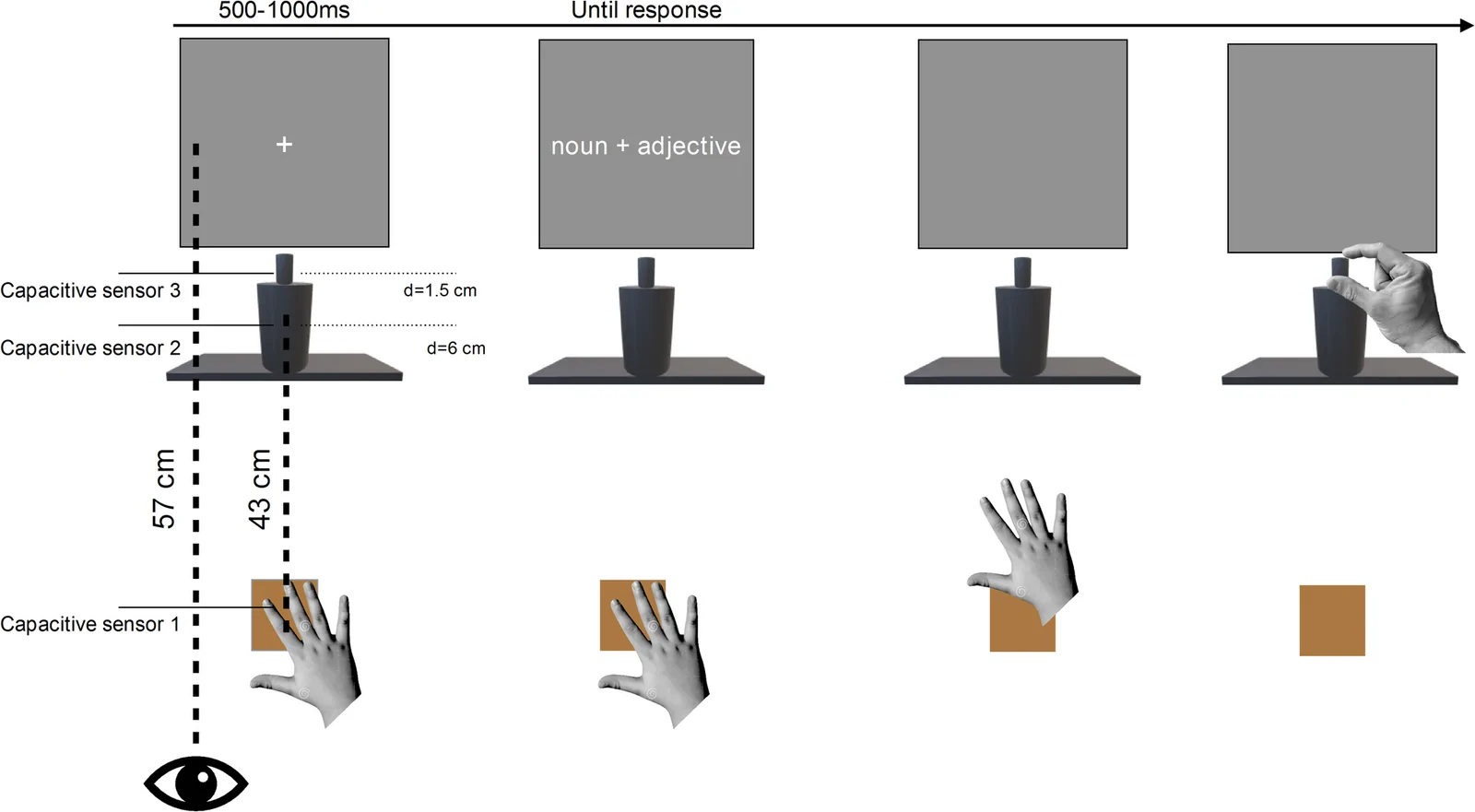
Representation of the experimental setup and the sequence of events

The experiment took place in a sound-attenuated and dimly illuminated room. The experimental apparatus consisted of a 24′′ monitor connected to a computer running E-Prime 2.0 software. The viewing distance was fixed at 57 cm by using an adjustable head- and chin-rest placed in front of the screen. The response device consisted of three parts (see Fig. 1): two wooden cylinders placed on top of each other and a squared starting base (10 cm × 10 cm). The dimension of the bottom cylinder was compatible with a power grasp (power cylinder: h = 14 cm, d = 6 cm), while the dimension of the top cylinder was compatible with a precision grasp (precision cylinder: h = 4 cm, d = 1.5 cm). The cylinders were placed 43 cm from the chin-rest. The starting base was located centrally with respect to participants’ body midline (see Fig. 1). All three parts of the response device were connected to an external USB device trigger through three separate capacitive sensors. The three device parts allowed us to measure the Reaction Times (RTs) and the Movement Times (MTs) for precision and power reach-to-grasp movements (Bub & Masson, 2010; Makris et al., 2011; Symes et al., 2008). The RTs and the MTs are related to the planning and the execution (or actualization) of the movement, respectively (Castiello, 1999, 2005; Glover, 2004; Jeannerod, 1984). Each trial started when participants placed the palm of their right hand on the starting base. The device did not require pressure to detect the presence of the hand. In this way, it was possible to avoid the activation of hand or arm muscles, reducing possible motor interference between the action required to press a key and the response action. When participants placed their right hand on the starting base, the fixation cross (bold courier new, 30-point size) appeared in the centre of the screen. To avoid habituation the fixation cross remained on the screen for a variable duration, randomly selected between 500 and 1000ms. After this variable interval, two words (the noun associated with an adjective) replaced the fixation cross and remained on the screen until the hand was lifted from its resting position. Both adjective and noun appeared on the same string in the center of the screen (1920 × 1280 resolution) in white characters on a black background (bold courier new, 24-point size).

Stimuli included thirty-two Italian object nouns. Sixteen nouns referred to natural objects, eight graspable with a precision grip (e.g., oliva, olive) and eight with a power grip (e.g., ‘patata’, potato). Sixteen nouns referred to artifacts, eight graspable with a precision grip (e.g., ‘chiodo’, nail) and eight graspable with a power grip (e.g., ‘bottiglia’, bottle). On 256 trials, the noun was combined with one adjective selected from four possible ones and denoting the spatial position of the object (near vs. far). The adjectives were: “vicino”, “prossimo” for Near condition and, “distante”, “lontano” for Far condition. Each noun-adjective combination was repeated randomly and with equal probability (four times). On 32 trials, the noun was followed by an adjective referring to human qualities (e.g., ‘oliva emotiva’, emotive olive). These noun-adjective combinations were randomly presented and repeated two times.

### Procedure

Participants were asked to categorize a noun, presented on the screen with an adjective, as natural or artifact. They had to perform a right-hand precision or power reach-to-grasp movement toward a custom-made response device (see Fig. 1). Grasp movements were either compatible or incompatible with the ones normally used to manipulate the objects denoted by the noun (e.g. precision grasp compatible with olive but incompatible with bottle). Half of the participants categorized nouns as natural by performing whole-hand (power) reach-to-grasp movements and as artifact by performing index-thumb (precision) reach-to-grasp movements. The remaining half of the participants followed the opposite category-response mapping. When the object noun was presented with an adjective denoting a spatial position, participants had to perform the categorization (*reach trials*). Participants were instructed to refrain from performing any reach-to-grasp movement and to lift their right hand when the noun-adjective combination was meaningless *(catch trials)*, that is the adjective denoted a human quality (e.g., arrogant cup). Catch trials were randomly presented during the task to ensure that participants read the whole noun-adjective combination. On all trials (both reach trials and catch trials), the stimuli disappeared from the screen when the participants lifted their hand.

The experimental task (288 trials in total) started after a practice session of 40 trials. Participants were tested individually, and they were instructed to find the best compromise between speed and accuracy while performing the task.

### Analyses

Participants’ error rate, RTs (ms), and MTs (ms) were recorded and analyzed. We excluded from the analyses practice and catch trials. The RTs were measured from stimulus onset until the moment in which participants lifted their hand from the starting base. The MTs were defined as the difference between the time in which participants completed the reach-to-grasp movement (the grasp of one of the two cylinders) and the time in which they lifted their hand from the starting position.

Correct RTs and MTs data were modeled after removing anticipatory responses (RTs < 150ms). The remaining distributions of RTs were inspected to evaluate deviations from normality. As these distributions are typically characterized by positive skewness, we employed an iterative Box–Cox procedure to search for the meaningful lambda transformation parameter (Box & Cox, 1964; Klein Entink et al., 2009) that best yielded a reduction of skewness across participants. After that, if visual inspection of the residuals still showed a marked deviation of the sample quantiles distribution from the theoretical quantiles, we assessed whether these values should be considered as outliers, using the following criterion:

$$\:\frac{Residual\left(RTs\right)\:-\:Median\:of\:residual\left(RTs\right)}{Median\:absolute\:deviation\:of\:residual\:\left(RTs\right)}\:$$

We excluded from the analysis responses that exceeded 3 times the criterion (Leys et al., 2013).

Given the design of the experiments with multiple observations for participants and stimuli, we modeled the data using the mixed-model approach. All models presented in the study included the fixed effects of Object Category (2 levels: natural and artifact), Compatibility (2 levels: compatible and incompatible) and Spatial Adjectives (2 levels: Near and Far), as well as all their interactions. Participants and Stimuli were set as random effects (Barr et al., 2013). Trials errors were modeled using logistic regression, while RTs and MTs were modeled using liner mixed models (LMM). All models were computed using R 4.2.0 (R Core Team, 2022), using lmer() function from lme4 package (Bates et al., 2015). We computed 95% CIs discounting participant-by-participant variation in order to interpret intervals that do not overlap as evidence of a difference between conditions.

Previous evidence suggested that the GCEs related to sensorimotor processing need time to emerge when objects are verbally as well as visually presented. GCEs tend to be larger with slower responses, due to a deeper sematic processing of the items (i.e., last bins of the bin analysis; Ambrosecchia et al., 2015; Bub et al., 2018; Bub & Masson, 2012; Garofalo et al., 2020, 2022). Given this temporal signature of the effect, we applied the Vincentization procedure first described by Ratcliff (1979) to examine whether similar distributional patterns could emerge in the semantic processing of verbal stimuli. For each participant and condition, correct RTs were rank-ordered from fastest to slowest and divided into quartiles: Bin 1 (0–25th percentile), Bin 2 (26th–50th percentile), Bin 3 (51st–75th percentile), and Bin 4 (76th–100th percentile), with each bin containing exactly 25% of trials. Mean RTs were then calculated for each bin across participants within each condition, creating Vincentized distributions that preserve distributional shape while controlling for individual baseline differences. This approach provides a descriptive proxy for temporal dynamics, offering a valuable insight into whether the GCE evoked by object nouns could be modulated by the presence of spatial words.

### Results

#### Errors 

The error rate data showed that participants made categorization errors on 4.95% of the total number of trials, 4.21% on *reach* trials and the remaining 0.74% on *catch* trials. Individual participants showed error rates ranging between 0.7% and % 10.3 of all trials.

The model did not show any reliable main effect or interaction.

#### Reaction times

Thirty-five data points were removed across all subjects from the dataset as likely anticipatory responses (0.57%). Box-Cox transformation results in a Lambda=-0.5. Accordingly, the reciprocal of RTs (1/RTs) was chosen as an appropriate transformation for RTs data. Outliers’ detection procedure produced a loss of 1.35% (79 datapoints) of the data. Transformed RTs were submitted to LMM.

The model revealed the main effect of Grasp-Compatibility (t(23)=|2.94|, *p* = .007), with faster responses on compatible (M=905ms; SD = 110.95; SEM = 22.64) than incompatible trials (M = 932; SD = 114.34; SEM = 23.34). The interaction of interest between Grasp-Compatibility and Spatial Adjectives was not significant (t(23)=|0.89|, *p* = .36).

#### Bin analysis on RTs

To compare the distributions of RTs as a function of individual response speed, we binned the RTs as described in the analysis section. Data were submitted to a linear mixed model including Grasp-compatibility, Spatial Adjectives and Bins and all their interactions as fixed effects, while Participants and Stimuli were set as random effects. Results revealed statistically reliable interactions between Grasp-compatibility and Spatial Adjectives (t(23)=|3.21|, *p* = .001), Grasp-compatibility and Bins (t(23)=|3.52|, *p* = .0004) and the critical interaction Grasp-compatibility x Spatial Adjectives x Bins (t(23) = 4.66, *p* < .0001). Descriptive statistics are reported in Table 1. To assess the statistical reliability of the GCE within each bin, we performed a series of t-tests against 0. Results are reported in Table 1. Pairwise comparisons were carried out to assess differences between the GCE emerged with the near and far adjectives in each bin. In the first two bins, no significant differences emerged between the far and near conditions GCEs (ts < |1.20|, ps > 0.4). A trend emerges in the third bin (t=|1.45|, *p* = .08), and a significant difference was observed in the fourth bin (t=|3.55|, *p* = .009).

**Table 1:** Experiment 1 averages (ms) with associated standard errors (in parenthesis) of each bin are reported

		Bins			
		1	2	3	4
Near	Compatible	727 (16.9)	832 (18.2)	936 (22.8)	1130 (30.9)
	Incompatible	742 (17.2)	866 (19.1)	985 (24.2)	1199 (30.4)
	GCE	15*	34*	49*	69*
Far	Compatible	733 (15.8)	844 (18.6)	951 (22.4)	1174 (33.5)
	Incompatible	761 (18.7)	878 (21.2)	980 (24.9)	1156 (31.7)
	GCE	28*	34*	29*	-18

Grasp-Compatibility Effect (GCE) refers to the RTs difference between incompatible and compatible trials

Asterisks referred to significant t test against 0 (ts > |2.5|, *ps *< 0.02)

#### Movement times

Box-Cox procedure resulted in a Lambda = 0, hence MTs distribution was transformed in the logarithmic of MT. Log(MTs) were submitted to LMM. The model revealed a significant main effect of Grasp-Compatibility (t(23)=|3.35|, *p* = .0008), with faster reaching movement for compatible trials (M=352ms; SD = 89.92; SEM = 18.36) compared to incompatible ones (M=363ms; SD = 88.10; SEM = 17.98) and the main effect of spatial adjective (t(23)=|2.62|, *p* = .009), with faster reaching movement for far adjectives (M=355ms; SD = 90.80; SEM = 18.53) than for near ones (M=360ms; SD = 87.22; SEM = 17.81).

### Discussion

**Fig. 2:**
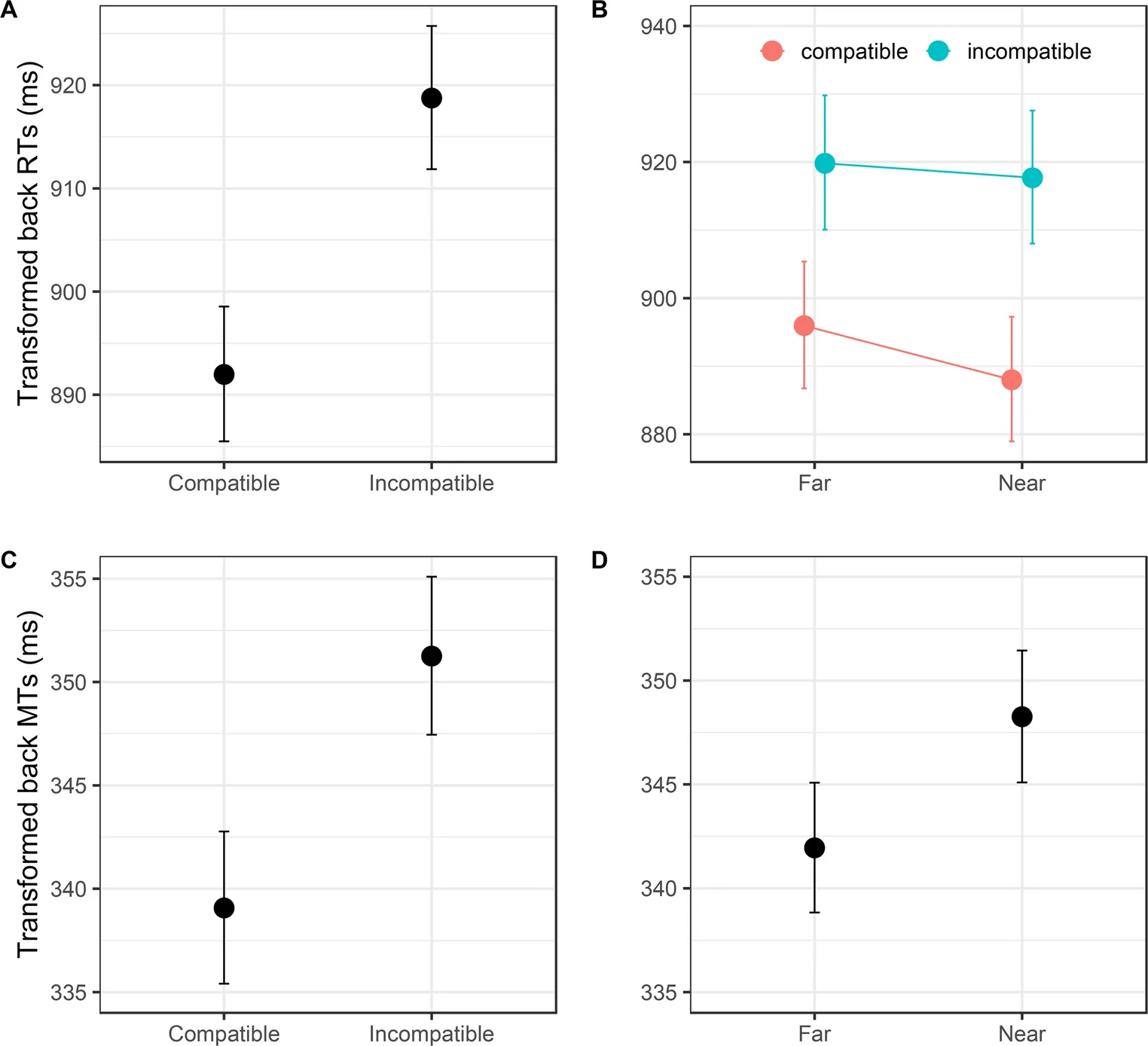
RTs and MTs estimated effects (transformed back to ms) are shown in the plots. Panel **A** and **C** report data as a function of the Grasp-Compatibility factor. In Panel **B**, the RTs interaction between Grasp-Compatibility and Spatial Adjective factors is depicted. Finally in Panel **D** the main effect of Spatial Adjective factor on MTs is represented. All error bars are 95% CIs, discounting participant-by-participant variation. Intervals that do not overlap can be interpreted as evidence of a difference between conditions

The present experiment investigated whether the grasp-compatibility effect elicited by graspable object nouns is modulated by adjectives specifying the location of objects (e.g., near vs. far). Results showed reliable GCEs elicited by object nouns both in the planning phase of the movement (RTs) and in the execution phase (MTs). In line with previous studies on affordances elicited by nouns (for reviews see Buccino et al., 2016; Vigliocco et al., 2011), this finding provides further evidence that the comprehension of object nouns involves the motor system. It has been proposed that the motor system is causally involved in the semantic processing of action-related words, such as nouns of graspable objects (e.g., Carota et al. 2012; Gough et al. 2012; Moseley and Pulvermüller 2014; Sato et al. 2008; Visani et al. 2022a, b). Language comprehension re-enacts mechanisms and processes of perception and action, without exactly reproducing them (Anderson, 2010; Gallese, 2008). This re-enactment takes place by virtue of mental simulation processes of experiential traces in the brain (Barsalou 2008; Borghi and Barsalou 2021; Buccino et al. 2016; Visani et al. 2022a, b). The interaction with object (or events in general) shapes these experiential traces embedding the words used to denote the object. As a result, when processing the word without its referent, the corresponding experiential trace gets reactivated, allowing the comprehension of the word itself (Buccino et al., 2016; Lachmair et al., 2016). The research question of interest concerned the modulation of this grasp-compatibility effect by spatial adjectives. Results of Experiment 1 were not conclusive in this respect. Even though the grasp-compatibility effect observed with near adjectives was somehow numerically larger than that observed with far adjectives (see Fig. 2B), the interaction between spatial adjective and grasp-compatibility effect) was not statistically reliable in the general analysis. At first, this observation may suggest that variable affordances such as the object location, even if explicitly specified by the presence of the adjectives, were not integrated in the sensorimotor representation evoked by graspable object nouns.

**Fig. 3:**
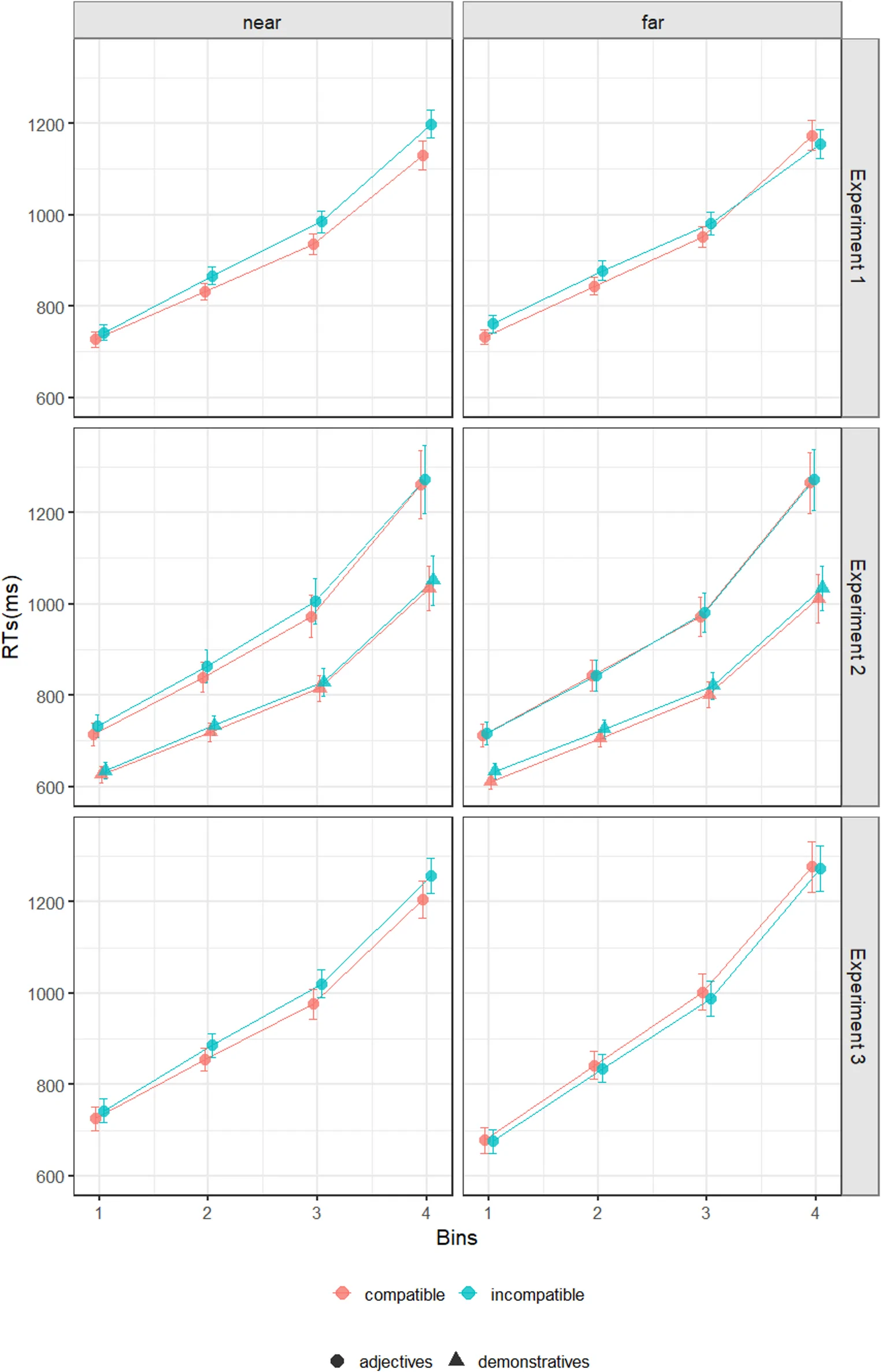
Results of Bin analysis for each experiment. All points represent the average values in each bin, for compatible and incompatible trials. Error bars indicate the standard error of the mean

However, indications supporting a differential impact of ‘near’ and ‘far’ adjectives came from the Bin analysis (see Fig. 3 – upper row). While reliable GCE were observed across bins for both near and far adjectives, the effect elicited with near adjectives was larger than the one observed with far adjectives in the last bins. This observation suggests that the processes responsible for the modulation of the compatibility effects with near and far adjectives were different to certain extents. It has been reported that grasp-compatibility effects related to sensorimotor processing need time to emerge when objects are visually as well as verbally presented and tend to be larger with slower responses (i.e. last bins of the bin analysis; Ambrosecchia et al., 2015; Bub et al., 2018; Bub & Masson, 2012; Garofalo et al., 2020, 2022). In principle, if the semantic processing of linguistic stimuli mimics the experiential response of interaction far adjectives should suppress the possibility to interact with objects, placing an object (i.e., the noun) in a non-reachable position (Cardellicchio et al., 2011; Costantini et al., 2011). This should lead to a reduction or disappearance of the grasp-compatibility effect with slower responses. The difference observed in the present experiment between GCE elicited by near and far adjectives with slower responses may indicate a reduction of the sensorimotor activation elicited by the noun when the adjective places the object beyond reachable space. If this is the case spatial adjectives are integrated with the object noun and modulate the sensorimotor simulation processes elicited during noun processing.

In favor of a sensorimotor interpretation of the effect of the adjectives there is also the effect detected in the MTs. MTs showed faster reaching movements when adjectives denoting far spatial positions were paired with nouns. This result is consistent with pivotal kinematic evidence (Gentilucci et al., 2000) in which participants had to grasp wooden blocks on which spatial adjectives are printed (“vicino”, near vs. “lontano”, far). The blocks were located within PPS in two different positions (nearer and farther). Results showed that participants moved faster their arm when “lontano” (farther) was printed on the block and this effect became stronger when the object was in far position. Accordingly, we can argue that spatial object properties specified by the adjective can affect also the execution of the movement (MTs) in addition to the planning phase (Jeannerod et al., 1995).

Considering together the numerical differences in the grasp compatibility effects between the two categories of adjectives, as well as the differences in the time course and movement times, it is possible that we are incurring in a Type II error whereby the modulatory effect related to the sensorimotor integration between adjectives and nouns is actually present but not clearly detected by the statistical analyses. For this reason, we decided to run a conceptual replication of Experiment 1 introducing a new experimental condition (Spatial Demonstratives) and reducing the number of spatial adjectives to the ones more frequently used in the Italian language (‘Vicino’ vs. ‘Lontano’).

## Experiment 2

The aim of Experiment 2 was twofold. First, we sought to clarify results of Experiment 1, in which the modulation of the grasp-compatibility effect by spatial adjectives was only statistically observed when the time course of the effect was considered. Hence, to disentangle between the possibility of a Type II error or the actual absence of sensorimotor integration between nouns and spatial adjectives, we replicated Experiment 1 with a new pool of participants. In this experiment participants completed two tasks. The spatial adjective (SA) task was virtually identical to the task described in Experiment 1 with the exception that only two spatial adjectives (the more frequent ‘vicino’ and ‘lontano’, near and far, respectively) were included in the task. If the spatial information conveyed by the adjective is sufficient to modulate the sensorimotor activation elicited by the noun, a grasp-compatibility effect is expected when the noun is followed exclusively by the adjective ‘near’ indicating the presence of the object within PPS, similarly to what observed with objects visual images.

In addition, we investigated the effect of a different class of words denoting the spatial relation between the agent and the objects on the GCE, that are spatial demonstratives used in their adjectival function. Spatial demonstratives should anchor objects distance relative to the speaker and are inherently linked to action through their association with pointing gestures, making them the most extensively studied in a variety of context (Brovold et al., 2012; Caldano & Coventry, 2019; Coventry et al., 2008, 2014; Kemmerer, 1999; Peeters et al., 2021; Rocca et al., 2019; Rubio-Fernandez, 2022), but not yet associated with sensorimotor effects evoked by nouns of graspable objects. Given the intrinsically motor nature of these words, it is therefore interesting to investigate whether and how they are able to modulate the GCE. Moreover, the different syntactic positions of these word classes in Italian (post-nominal adjectives vs. pre-nominal demonstratives) allows us to test whether the relative position of spatial words affects the GCE evoked by object nouns, thus providing a more complete picture of how language interfaces with the sensorimotor processes.

To this aim, we asked participants to complete the spatial demonstrative (SD) task in addition to the SA task. In this grasp-compatibility task, the spatial demonstrative ‘this’ (‘questo’ in Italian) and ‘that’ (‘quello’) were presented instead of the spatial adjective ‘near’ (‘vicino’) and far (‘lontano’) and preceded the object noun. Similarly to what hypothesized for spatial adjectives, a grasp compatibility is expected only when the object noun is combined with ‘*this’*, that is when the spatial information conveyed by the spatial demonstratives specifically places the objects within reachable space.

## Method

### Participants

Twenty-eight new participants (20 identifying as female; 6 identifying as male; mean age in years = 22.4 ± 1.47) volunteered to take part in the experiment at the University of Bologna. Two participants were excluded as they were left-handed; the remaining 26 participants were right-handed, as measured by a standard handedness inventory (Oldfield, 1971), native-Italian speakers, and had normal or corrected-to-normal vision. All of them were 18 years old, or older, at the time of the study and naïve as to the purpose of the experiment. Participants gave their informed consent before participation, following the ethical standards of the Declaration of Helsinki. The study was approved by the local ethical committee (approval number: 0026467).

### Apparatus, stimuli and procedure

The experimental apparatus was the same of Experiment 1 with the following exceptions. The number of spatial adjectives was reduced from four to two, to match the number of adjectives with the two demonstratives. We select the adjectives with the higher semantic frequency (i.e., “Vicino” – near; “Lontano” – far). We tested this task (Spatial Adjective, SA) with the task in which spatial demonstrative (i.e., “Questo” – this; “Quello” – that) were combined with graspable object nouns (Spatial Demonstrative, SD). In the Italian language, adjectives are typically presented after the noun, but demostratives are presented before the noun. To maintain the canonical word order, in the SA task the adjective was presented after the noun, whereas the demonstrative was presented before the noun in the SD task. Both the SA and SD tasks consisted of one block of 256 trials.

We modified catch trials (32 trials for each task) to make them comparable under the different experimental conditions (SA and SD). To this aim, on catch trials a pseudoword (e.g. “quispo”) was presented with the object name. The pseudoword followed the noun in the SA task and preceded the noun in the SD task. All object nouns used in this experiment were the same used in Experiment (1) In Experiment 1, we combined nouns with emotional adjectives. The adjective was presented after the name, in line with the typical presentation order in the Italian language. This was not viable for Experiment (2) Because demonstratives typically precede the noun in Italian, presenting an adverb followed by an emotional adjective would create a syntactically anomalous sequence in the SD task. Therefore, we opted for pseudowords in both the SA and SD tasks of Experiment 2. In these tasks, catch trials are crucial to ensure that participants fully processed both words (noun + adjective in the SA task, demonstrative + noun in the SD task) on each trial before executing or withholding the reach-to-grasp response. This prevented that participants responded based solely on the task-relevant object noun, ensuring that they processed semantically the complete spatial-noun combination. Importantly, the use of pseudowords allowed us to have the same stimulus on catch trials in both tasks without introducing confounding syntactic violations due to other specific word categories.

Participants were instructed to categorize the noun as natural or artifact, by performing either a power or a precision grip toward the manipulanda. They were instructed to read also the word preceding or following the noun and to refrain from responding in case this was a pseudo-word. Each participant completed both the SA and SD tasks. Task order was counterbalanced across participants as well as response mapping. Between Tasks a resting break of 20 min was provided.

### Analysis

The general procedure for data treatment and analysis was the same as the one described for the previous study, with the following exceptions. The models included the fixed effects Compatibility (2 levels: compatible and incompatible), Space (2 levels: Near and Far) and Task (2 levels: Spatial Adjectives vs. Spatial Demonstratives), as well as all their interactions.

Participants and Stimuli were set as random effects nested in the presentation orders (Barr et al., 2013). RTs and MTs were modeled using LMM. Analyses were conducted with R 4.2.0 (R Core Team, 2022), using lmer() function from lme4 package. Planned t-test was carried out between reliable grasp-compatibility effect in the SA and SD conditions. Finally, the bin analysis was also performed to investigate possible temporal differences in the evolution of the grasp-compatibility effect between the two tasks.

### Results

#### Errors

The error rate data showed that participants made an average of 2.80% categorization errors across the total number of trials (2.19% on *reach* trials and 0.61% on *catch* trials). Individual participants showed error rates ranging between 0.2% and 9.6% of all trials. Given the low error rate, these were not analyzed further.

#### Reaction times

Ninety-four data points were removed from the dataset as likely anticipatory responses (0.75%). Box-Cox transformation results in a Lambda=-1. Accordingly, also in this experiment the reciprocal of RTs (1/RTs) was chosen to normalize the data. Outliers’ detection procedure resulted in the loss of 0.89% (108 datapoints) of the data.

**Fig. 4:**
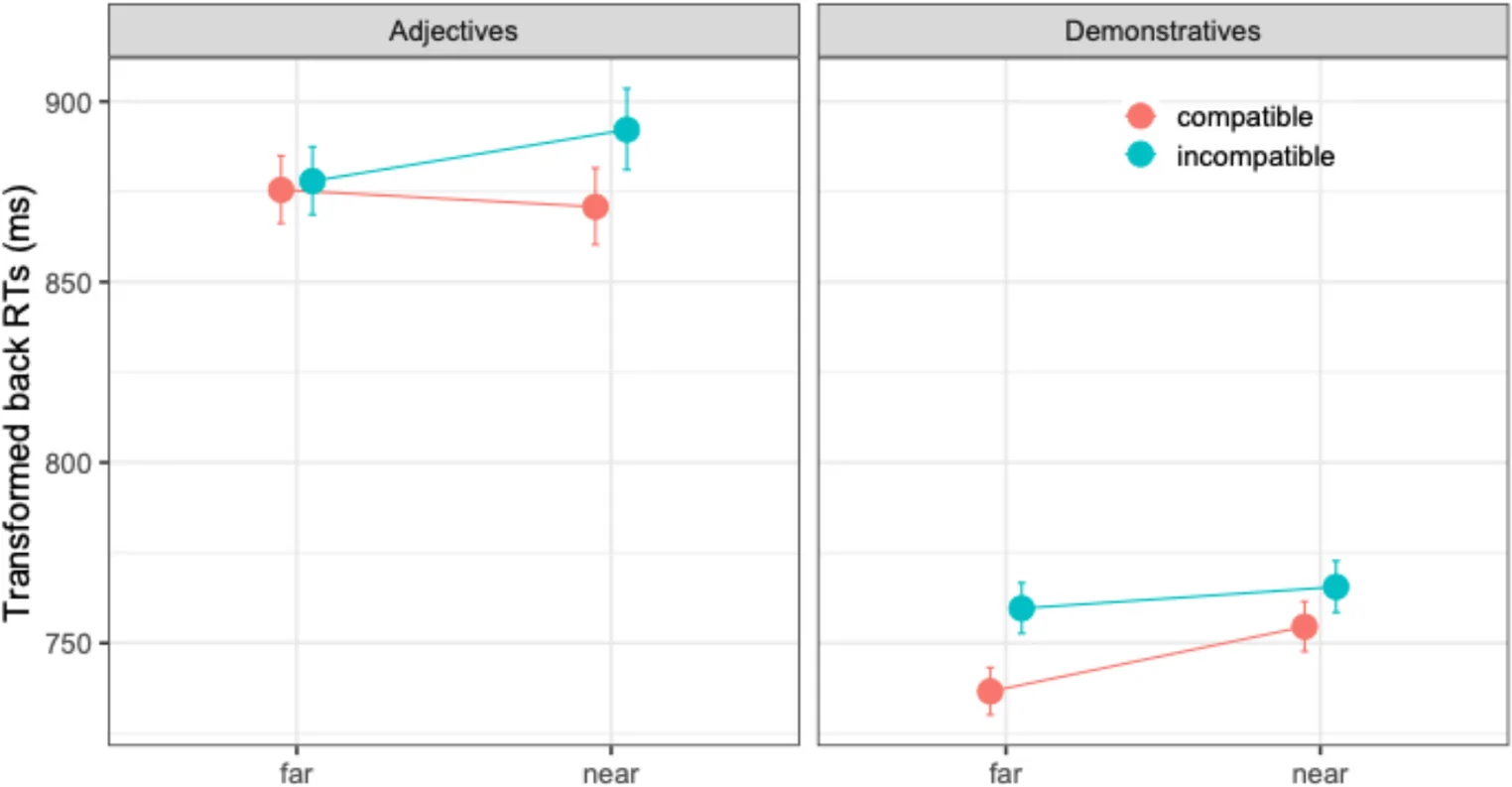
RTs estimated effects (transformed back to ms) are shown in the plots as a function of Word Category. All error bars are 95% CIs, discounting participant-by-participant variation. Intervals that do not overlap can be interpreted as evidence of a difference between conditions

**Fig. 5:**
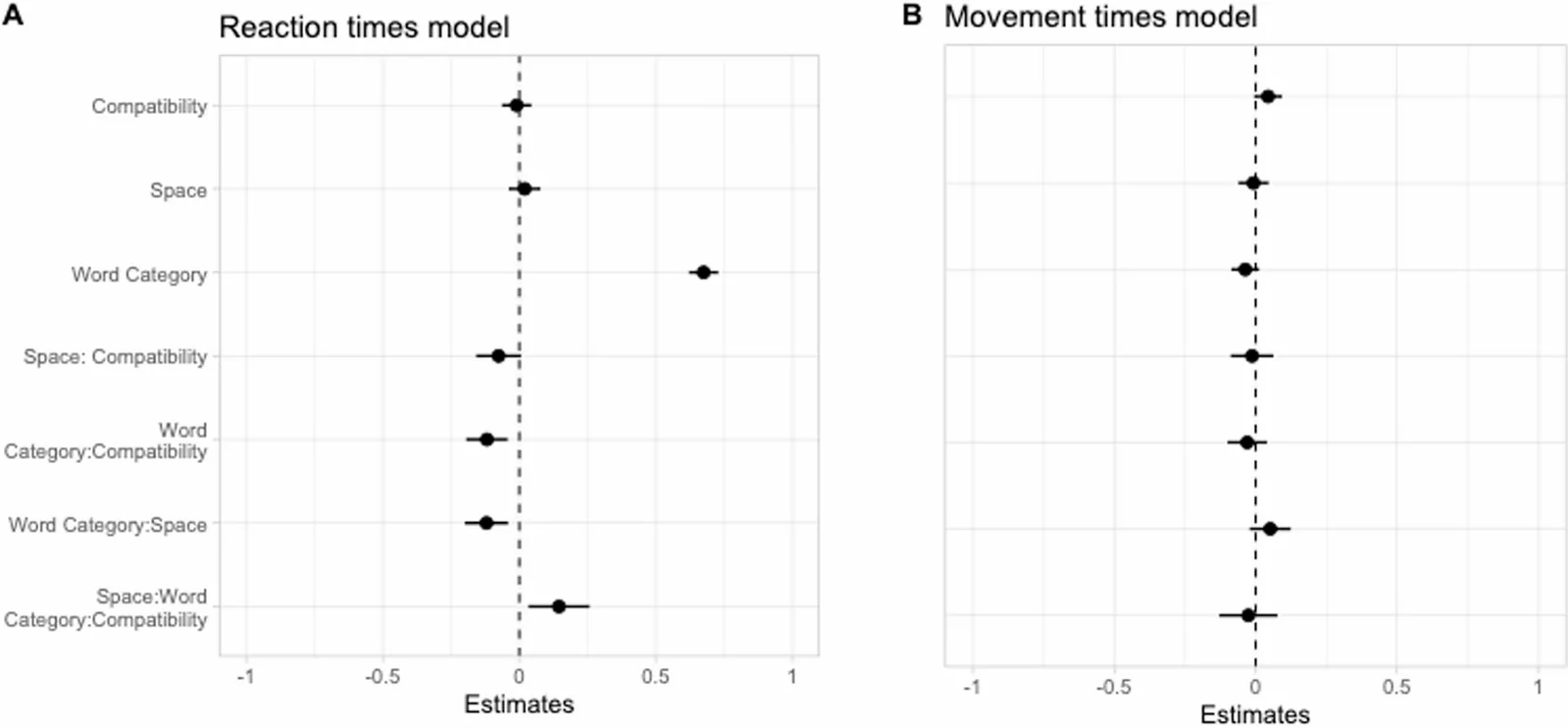
Forest plots for transformed RTs and MTs models of Experiment 2. On the x-axes are reported the estimates fixed effects. The dotted lines indicate the absence of the effect. The dots indicate the fixed effects estimated by the model with the associated 95% CIs

LMM revealed the main effect of Task (t(25) =|17.55|, *p* < .0001), with faster responses in the SD task (M=770ms; SD = 123.49; SEM = 24.22) than in the SA task (M=909ms; SD = 188.15; SEM = 36.89). The interaction between Grasp-Compatibility and Task (t(25)=|3.04|, *p* = .0024), as well as the interaction between Space and Task (t(25)=|2.17|, *p* = .0313) were statistically significant. Importantly, also the three-way interaction between Grasp-Compatibility, Space and Task was reliable (t(25)=|2.55|, *p* = .0109; see Figs. 4 and 5A). To further explore this interaction, separate linear mixed-effects models were fitted for the SA and SD tasks. In the SA task, the Grasp-Compatibility × Space interaction is significance (t(25) = |2.025|, *p* = .046). Holm-corrected follow up contrasts revealed a significant GCE with near spatial adjectives, with compatible trials faster than incompatible trials (t = 2.81, *p* = .029), whereas no GCE emerged with far spatial adjectives (t = 0.41, *p* = 1.00). No other difference emerged by these contrast (ts < 1.53, *p* > .62).

In the SD task, the Grasp-Compatibility × Space interaction was significant (t(25) = |2.12|, *p* = .032). Follow up contrasts with holm correction revealed a significant GCE with far spatial demonstratives (t = 4.77, *p* < .001), as well as a significant difference between compatible-far and compatible-near trials (t = 2.75, *p* = .024), whereas the GCE with near spatial demonstratives did only approach the significance (t = 2.16, *p* = .091). Descriptive statistics are reported in Table 2.

#### Bin analysis on RTs

Figure 3 - second row shows the time course of the GCE as a function of response times (bins), separately for each task (SD and SA) and each space condition (near and far). Results of the Bin analysis revealed that the interaction of interest between Grasp Compatibility, Space, Task and Bins was significant (t(25)=|2.01|, p = .045). Separate analyses carried out for the SA and SD tasks revealed reliable interactions between Grasp-compatibility, Space and Bins in the SA task (t(25) = 2.65, p = .014) but not in the SD task (t = 0.97, p = .380). To further explore the three-way interaction observed in the SA task, the GCE was calculated for each participant, bin and spatial adjective. To determine in which condition the GCE was reliable within each bin, we performed a series of t-tests against 0. Results are reported in Table 2. Pairwise comparisons were carried out between the GCEs observed with near and far adjectives separately for each bin. A trend toward significance emerged in the first bin (t=|1.39|, p = .09), followed by significant differences in the second and third bins (ts > |2.03|, ps < 0.037). No significant difference between near and far GCEs was detected in the fourth bin (t = 0.12|, p = .45).

#### Movement times

Box-Cox procedure resulted in a Lambda = 0, hence also in this Experiment, the MTs distribution was log transformed. Log(MTs) were submitted to LMM. The model failed to reveal any effect. Only the main effect of Grasp-Compatibility approached significance (t(25)=|1.74|, p = .0825; see Figs. 5B), with numerically faster reaching movements for compatible trials (M=359ms; SD = 83.22; SEM = 16.32) as compared to incompatible ones (M=361ms; SD = 82.59; SEM = 16.20).

**Table 2:** Experiment 2 averages (ms) with associated standard errors (in parenthesis) of each bin are reported.

			Bins			
			1	2	3	4
Adjectives	Near	Compatible	715(24.56)	840(33.65)	973(47.08)	1261(74.17)
		Incompatible	732(25.18)	864(35.29)	1006(50.15)	1273(74.50)
		GCE	16 *	24 *	32 *	11
	Far	Compatible	713(24.39)	843(33.28)	973(43.16)	1265(66.43)
		Incompatible	717(25.72)	843(33.91)	981(42.92)	1272(66.84)
		GCE	4	0	8	7
Demonstratives	Near	Compatible	627(17.54)	720(20.26)	816(28.21)	1034(49.07)
		Incompatible	635(17.67)	734(20.70)	829(30.81)	1052(54.38)
		GCE	8	13*	13	18
	Far	Compatible	611(16.37)	706(19.09)	802(28.87)	1012(52.38)
		Incompatible	634(17.00)	726(19.38)	821(29.34)	1035(48.39)
		GCE	23*	20*	20*	23

Grasp-Compatibility Effect (GCE) refers to the RTs difference between incompatible and compatible trials

Asterisks referred to significant t test against 0 (ts > |2.4|, *ps* < 0.04)

### Discussion

Results of Experiment 2 revealed that both adjectives and demonstratives specifying the location of objects in space were able to modulate the grasp-compatibility effect elicited by graspable object nouns. Importantly, in the *SA task* the adjective ‘near’ combined with a noun evoked a reliable grasp-compatibility effect, while no GCE emerged with the adjective ‘far’. This difference was observed in all bins (except the 4th ), as indicated by statistically reliable GCEs with near but not with far adjectives. These findings expand those reported in Experiment 1, demonstrating a reliable difference between the GCE with near and far adjectives. Interestingly, a reversed pattern of results was obtained in the *SD task.* Results revealed that the GCE was larger when the object noun was presented with ‘that’ (far location) compared to ‘this’ (near location).

In the Italian language, typically adjectives are presented after the noun whereas demonstratives are presented before it. To avoid violations of the correct syntactical order of words, in the SD and SA task the noun and spatial words were presented with an opposite order, that is noun + adjective in the SA task and demonstrative + noun in the SD task. Given this relevant methodological difference, it is worth noting that we found an integration between the two categories of words regardless of the order in which these were presented, confirming that the correct syntactic order is a key feature of the sensorimotor integrative processes (Garofalo et al., 2024). Given that the GCE is elicited by the object noun, these results suggested that the position of the noun is not critical to the sensorimotor integrative processes.

## Experiment 3

Results of Experiments 2 confirmed the modulatory role of spatial adjectives on the GCE. Specifically, no significant GCE emerged when the noun was paired with far adjective while a reliable GCE was observed with near adjective. Although a numerical GCE reduction for far compared to near adjectives was also observed in Experiment 1, a statistically reliable GCE was present with both. To establish which of these two result patterns should be expected in a similar task, a third Experiment was carried out to assess the reliability/replicability of the modulatory effect of spatial adjectives on the GCE. Experiment 3 was an independent replication of the Spatial Adjective task of Exp. 2, carried out in the absence of the Spatial Demonstrative task to avoid potential contextual carryover effects from one task to the other.

## Methods

### Participants

Twenty-six new participants (12 identifying as female; 14 identifying as male; mean age in years = 24.9 ± 5.25) volunteered to take part in the experiment at the University of Bologna. All participants were right-handed, as measured by a standard handedness inventory (Oldfield, 1971), native-Italian speakers, and had normal or corrected-to-normal vision. All of them were 18 years old, or older, at the time of the study and naïve as to the purpose of the experiment. Participants gave their informed consent before participation, following the ethical standards of the Declaration of Helsinki. The study was approved by the local ethical committee (approval number: 0026467).

### Apparatus, stimuli and procedure

The experimental apparatus and stimuli were identical to those of Experiment 2 – SA task.

### Analyses

The general procedure for data treatment and analysis was the same as described for Experiment 1 and 2.

### Results

#### Errors

The error rate data showed that participants made categorization errors on 3.61% of the total number of trials (210 data points). Given the low error rate, errors were not analyzed further.

#### Reaction times

**Fig. 6:**
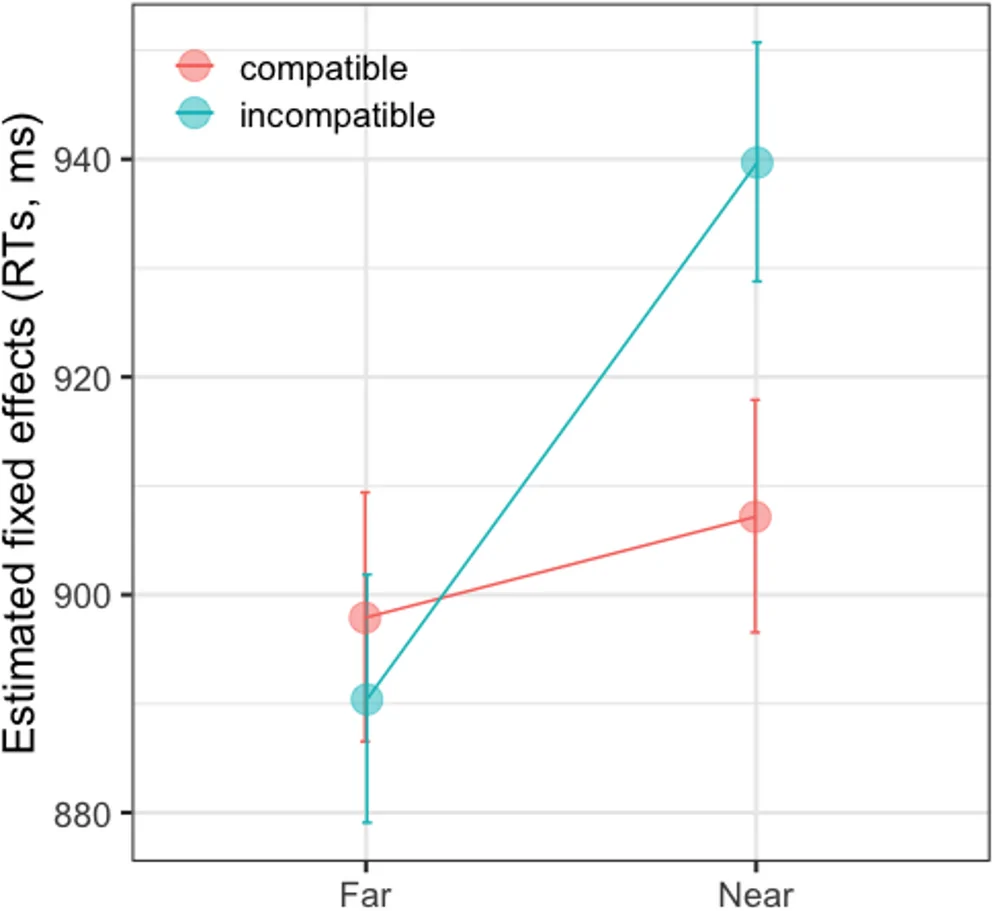
RTs estimated effects (transformed back to ms) are shown in the plots as a function of Space. All error bars are 95% CIs, discounting participant-by-participant variation. Intervals that do not overlap can be interpreted as evidence of a difference between conditions

Eighty-six data points were removed from the dataset as likely anticipatory responses (1.48%). Box–Cox transformation resulted in a Lambda = 0. Accordingly, the logarithm of RTs (log(RTs)) was chosen as an appropriate transformation for RTs data. The outlier detection procedure resulted in the loss of 1.02% (57 data points) of the data. Transformed RTs were submitted to a linear mixed model. The model revealed that the main effects of Spatial Adjective and Grasp-Compatibility were not significant (t(25)= |1.15|, p = .248; t(25)= |0.91|, p = .364, respectively). Crucially, the interaction between Grasp-Compatibility and Spatial Adjective was significant (t(25)= |3.48|, p < .001; Fig. 6). Follow up contrasts using Holm correction showed a statistically reliable GCE (34 ms; t = 4.16, p < .001) was evoked by graspable object nouns when paired with near adjectives, with compatible trials (M = 918 ms; SD = 152.46; SEM = 30.31) faster than incompatible ones (M = 952 ms; SD = 145.11; SEM = 28.87). By contrast, the GCE was not statistically significant for far adjectives (− 6 ms; compatible trials, M = 914 ms; SD = 177.76; SEM = 35.12; incompatible trials, M = 908 ms; SD = 172.12; SEM = 34.74; t = 0.91, p = .497). None of the other contrasts reached significance (ts < 1.15; ps > 0.110).

#### Bin analysis on RTs

To examine the time course of the GCE, RTs were binned following the Vincentization procedure described in Experiment 1. The LMM carried out on this data included Grasp-Compatibility, Spatial Adjective, and Bins as fixed effects, with all their interactions, and Participants and Stimuli as random effects. Results revealed a reliable main effect of Bins (t(23) = |78.88|, p < .0001), reflecting slower responses in later bins, as well as a main effect of Spatial Adjective (t(25) = |16.97|, p < .0001). The three-way interaction between Grasp-Compatibility, Spatial Adjective, and Bins was significant (t = |2.31|, p = .021), revealing that the modulation of the GCE as a function of response speed differed between spatial adjectives. Descriptive statistics are reported in Table X. To assess the statistical reliability of the GCE within each bin, we calculated the GCE separately for each participant, spatial adjective, and bin, and performed a series of t-tests against 0. Results are reported in Table 3. With near adjectives, statistically reliable GCEs were present in the 2nd (mean GCE = 31 ms; t(25) = 3.62, p = .001), 3rd (mean GCE = 44 ms; t(25) = 4.84, p < .001), and 4th bins (mean GCE = 52 ms; t(25) = 5.07, p < .001), but not in the 1st bin (mean GCE = 17 ms; t(25) = 1.61, p = .120). With far adjectives, GCEs were numerically negative in all bins (corresponding slower than non-corresponding responses) but never reached statistical significance (all |ts| ≤ 1.61, all ps ≥ 0.119). Pairwise comparisons with holm correction were carried out to assess differences between the GCE that emerged with near and far adjectives in each bin. Reliable differences between GCEs with near and far adjectives were present in all bins except the 1st (Bin 1: t(25) = 1.29, p = .210; Bins 2 to 4: ts(25) > 2.90; ps < 0.008).

**Table 3:** Experiment 3 averages (ms) with associated standard errors (in parenthesis) for each bin. The Grasp-Compatibility Effect (GCE) is calculated as the RT difference between incompatible and compatible trials. Asterisks refer to significant t tests against 0

		Bin 1	Bin 2	Bin 3	Bin 4
Near	Compatible	726 (25.90)	855 (25.82)	977 (32.74)	1205 (40.88)
	Incompatible	743 (26.52)	886 (26.46)	1021 (30.24)	1257 (38.53)
	GCE	17	31*	44*	52*
Far	Compatible	678 (28.00)	842 (30.50)	1003 (38.52)	1277 (55.55)
	Incompatible	676 (26.17)	835 (30.62)	988 (37.93)	1273 (50.66)
	GCE	-2	−7	−15	−4

Asterisks refer to significant t tests against 0

#### Movement times

Box–Cox procedure resulted in a Lambda = 0, hence the MTs distribution was log-transformed. Log(MTs) were submitted to a LMM. The model failed to reveal any significant effect. The main effect of Grasp-Compatibility approached significance (t = |1.70|, *p* = .089), with numerically faster reaching movements observed for compatible trials (M = 348 ms; SD = 88.72) compared to incompatible ones (M = 352 ms; SD = 86.31). Neither the main effect of Spatial Adjective (t = |1.09|, *p* = .274) nor the interaction between Grasp-Compatibility and Spatial Adjective (t = |0.07|, *p* = .942) were significant.

### Discussion

Experiment 3 provided a clear and independent replication of the modulation of GCE by spatial adjectives. Results of this Experiment demonstrated a statistically reliable Grasp-Compatibility × Spatial Adjective interaction in a standalone spatial adjective task. Results are extremely similar to those observed in Experiment 2 in which the SA task was administered with the SD task. Given the pattern of results observed across these three studies it is likely that the lack of grasp-compatibility interaction emerged in Experiment 1 reflected a Type II error. Overall, results confirm that spatial adjectives specifying the location of the object in PPS (near) versus EPS (far) are integrated into the sensorimotor representation elicited by graspable object nouns.

Consistent with Experiments 1 and 2, the bin analysis revealed a significant three-way interaction between Grasp-Compatibility, Spatial Adjective, and Bins, demonstrating that the time course of the GCE differed between near and far adjective conditions. As observed in the previous experiments, the GCE for near adjectives showed an incremental pattern across bins, characteristic of sensorimotor processing that requires time to emerge (Ambrosecchia et al., 2015; Bub et al., 2018; Bub & Masson, 2012; Garofalo et al., 2020, 2022). By contrast, the GCE associated with far adjectives did not show this incremental pattern, suggesting that adjective placing the object beyond reachable space through linguistic means reduced the engagement of the hand motor system.

## General Discussion

In three experiments, the impact of spatial information conveyed by different word categories on the grasp-compatibility effect elicited by graspable object nouns was investigated. In Experiment 1, adjectives denoting near and far objects’ positions were combined with graspable object nouns. Data showed a numerically larger GCE and an increasing pattern of GCE in the bin analysis for near compared to far adjective trials. These observations were replicated in Experiment 2 and 3, which showed significantly larger GCEs when the object noun was presented before a near compared to a far adjective.

Building on these adjective effects, Experiment 2 also investigated for the first time the modulatory impact of spatial demonstratives presented with object nouns on the GCE. Surprisingly, no GCE was observed when proximal position was indicated by ‘this’, but the effect was observed when the spatial demonstrative expressing distal position (i.e., ‘that’) was combined with the noun. We speculate that proximal demonstratives denote a location roughly equivalent to the position of the reader, i.e., the object is likely already within the agent’s hands, making redundant the activation of the grasping motor program.

### The effects of near and far adjectives

Spatial information conveyed by adjectives clearly modulated the grasp compatibility effects elicited by object nouns. As expected, when nouns of graspable objects are combined with near adjectives, the grasp-compatibility effect is reliable. This result is further supported by the bin analysis of Experiment 2, where the compatibility effects obtained with near adjectives increased in magnitude as participants’ response times increased, at least until the 3rd bin, paralleling the results of Experiment 1. Notably, this pattern of results mirrors findings reported when visual images of objects are used (Costantini et al., 2011). In visual perception studies, placing a graspable object in the extrapersonal space (EPS) reduces the involvement of the motor system since the object cannot be grasped and used. Our findings suggest that spatial adjectives function similarly by defining portions of spaces related to peripersonal space (PPS) and EPS through linguistic means. Indeed, presenting an object outside of reachable space, whether visually or linguistically, severely affects its ability to engage the hand motor system, as shown by reduced or absent affordance effects when objects were presented in EPS (Costantini et al., 2010, 2011).

This deep-rooted nature of spatial distinctions is further supported by extensive neuropsychological evidence documenting PPS/EPS dissociations in patients with neglect or crossmodal extinction (for review see di Pellegrino & Làdavas, 2015; Halligan et al., 2003). These patients show that deficits can occur selectively in near but not far space, and vice versa (e.g., Bisiach et al., 1986; Halligan & Marshall, 1991), suggesting that the contrastive nature in the use of near/far adjectives taps into fundamental differences between the sensorimotor mechanisms responsible for the encoding of PPS and EPS.

However, an intriguing question arises when comparing how spatial information affects sensorimotor processes in visual versus linguistic modalities. Systematic differences exist between sensorimotor processes elicited by object images and their verbal referents regarding spatial location. Ferri and colleagues (2011) demonstrated compatibility effects for object images only in PPS (see also Costantini et al., 2010), whereas when participants performed reaching tasks after matching nouns with object images, the compatibility effect extended to both PPS and EPS. This difference suggests that while visual objects elicit both stable and variable affordances, noun processing mainly includes stable affordances(Borghi & Riggio, 2009, 2015) that is, the pragmatic features related to manipulation without specific location information.

Our results demonstrate that this limitation of linguistic processing can be overcome through the use of spatial adjectives. When adjectives explicitly specify whether objects are near (likely reachable) or far (likely unreachable), this information becomes incorporated into the object’s sensorimotor representation. What makes this finding particularly compelling is that despite spatial adjectives not specifying well-defined positions but only sectors of space, the underlying distinction between PPS and EPS appears so deep-rooted in the brain’s sensorimotor circuits that even this broad near/far distinction is sufficient to affect sensorimotor processes. To understand the mechanism underlying these adjective effects, we can turn to the Linguistic Focus Hypothesis (Taylor & Zwaan, 2008; Zwaan et al., 2010), which proposes that linguistic constituents from specific grammatical categories are more effective than others in situating the simulation during language comprehension. Converging evidence has shown that the grasp-compatibility effect elicited by object nouns is selectively modulated by different categories of adjectives (Garofalo et al., 2021, 2024). For instance, when the object noun is presented with an adjective denoting a property preventing the possibility to grasp the object (e.g., broken), the compatibility effect was significantly reduced or reversed. Similarly, when combined with color adjectives, a compatibility effect was observed for natural but not artifact objects. Results of the present study complement and expand this body of work by demonstrating that also spatial adjectives modulate the GCE, by placing the object within or outside reachable space and thus specifying the actual feasibility of the grasp (see also Garofalo et al., 2026 for similar results).

Inspection of the response patterns of Experiments 2 and 3 indicates that the GCE modulation by spatial adjectives was primarily driven by incompatible trials. Data showed that compatible responses were comparable across near and far adjectives, whereas incompatible responses were systematically slower with near adjectives. This pattern of results may suggest that integrating *near* spatial information with the noun amplifies the cost of selecting a mismatching motor program on incompatible trials. Alternatively, it may indicate the absence of the expected cost associated with an incompatible response with *far* adjectives. However, the absence of a neutral baseline prevents the possibility to disentangle these options. Future studies including a neutral baseline could investigate whether this pattern reflects selective changes (driven by facilitation, inhibition or both) on trials with near and far adjectives (Littman & Kalanthroff, 2022).

From a neuroanatomical perspective, these modulatory effects of adjectives can be understood through the organization of the dorsal stream, which has been classically associated with the parametrization of actions. This stream elaborates visual properties to feed information to the motor cortex (Milner & Goodale, 2008; Rizzolatti & Matelli, 2003). Crucially, within this network, spatial information (variable affordances) is represented more dorsally compared to stable affordances, which are represented more ventrally (Borghi and Riggio 2015; Sakreida et al. 2016). This anatomical organization has important implications for understanding how linguistic processing engages motor systems. The circuits involved in stable affordances are reactivated during object noun processing, and when adjectives specify further object properties, these properties could be re-enacted by the same brain areas involved in experiencing interaction with the real object. Consequently, sensorimotor processing of noun-adjective combinations could engage the ventral part of the dorsal stream, affecting manipulative components of grasping (e.g., grasp-compatibility effect), while variable affordances expressed by spatial adjectives could be re-enacted more dorsally, mainly modulating reaching components.

### The effects of this and that

While adjectives provide explicit spatial information through descriptive terms, spatial demonstratives represent a different linguistic mechanism for encoding space. At first glance, spatial demonstratives could mirror the functional organization of PPS and EPS, with “this” associated with near, reachable locations and “that” with far, non-reachable ones (Caldano & Coventry, 2019; Coventry et al., 2008; Coventry & Diessel, 2025). However, recent evidence suggests that demonstrative selection depends on the possibility of agent-object interaction rather than on spatial distance per se (Coello & Bonnotte, 2013; Gervasi et al., 2024; Rocca et al., 2019). Notably, Gervasi et al. (2024) demonstrated that Italian speakers preferentially use “this” to refer to objects they physically control, even in extrapersonal space, suggesting that the proximal demonstrative encodes agent control over a referent rather than mere spatial proximity. Despite this established connection between demonstratives and spatial processing, no study had previously investigated the impact of spatial demonstratives when combined with graspable object nouns and grasping actions. Surprisingly, results revealed a pattern opposite to that observed with adjectives: a reliable GCE emerged for nouns presented with “that,” but not with “this.”

This dissociation can be interpreted through the theoretical framework proposed by Brovold and Grush (2012), which posits that demonstrative selection reflects the agent’s physical control over referents rather than distance-based space segments (see also Gervasi et al., 2024). Under this account, processing “this object” might place the object as already under the agent’s control, the proximal demonstrative denoting a state in which the object is effectively possessed, thus making the activation of grasping motor programs redundant. Conversely, the GCE observed with “that” may reflect the open possibility of interacting with the object, either directly through the agent’s own reaching and grasping, or indirectly when receiving an object from another agent.

If this interpretation is correct, spatial demonstratives are not grounded in a quantitative segmentation of space defined by arm’s reach. Instead, consistent with their deictic nature (Levinson, 1997), they may enable the agent to cover unlimited spatial distances depending on pragmatic context (Kemmerer, 1999; Talmy, 1983). A promising way to further test this account would involve cross-linguistic comparisons between languages with binary and ternary demonstrative systems, such as Spanish, which distinguishes between este (near the speaker), ese (near the listener), and aquel (far from both), potentially allowing dissociation between agent control and spatial distance.

## Conclusion

The present study corroborates the close involvement of sensorimotor systems in language processing while revealing important distinctions in how different word categories define spatial information. Spatial information specified by adjectives paired with object nouns modulated performance as shown by different motor parameters, revealing a hand-object metric of space analogous to that proposed for object images. By contrast, effects of spatial demonstratives presented with object nouns suggest that this word category does not simply map the contrast between PPS and EPS. Instead, this category of spatial words appears to be grounded in communicative contexts and characterized by high pragmatic flexibility.

These different patterns of GCE modulations observed for spatial adjectives versus spatial demonstratives suggest distinct roles of these word categories in defining agent-object possible interactions, with adjectives mapping stable spatial regions and demonstratives encoding dynamic aspects of object control and possession. Together, these findings advance our understanding of how language interfaces with motor systems and highlight the sophisticated ways in which different linguistic devices capture the complexity of spatial cognition.

## Data Availability

The datasets generated during and/or analyzed during the current study are available in the Figshare repository: 10.6084/m9.figshare.23301743.

## Code availability

The codes used to analyze data are available from the corresponding author on reasonable request.
